# New Approaches to Filling the Gap in Tuberculosis Drug Discovery

**DOI:** 10.1371/journal.pmed.0040293

**Published:** 2007-11-06

**Authors:** Martina Casenghi, Stewart T Cole, Carl F Nathan

## Abstract

For the first time in decades, say the authors, there is a tuberculosis drug pipeline, but the paucity of candidates is still cause for alarm.

Early stage drug discovery is a key bottleneck in the pipeline to find novel drugs for tuberculosis (TB) [1,2]. For diseases that affect people in wealthy countries, pharmaceutical companies actively scout advances in basic research in search of new and potentially lucrative drug targets. For TB, this is not the case: of the 1,556 new chemical entities marketed worldwide between 1975 and 2004, only three were for TB [3]. The general problem of antibiotic research and development (R&D) has been described elsewhere [4,5], but TB is worth singling out: it is the leading cause of death from bacterial infection, it is spread person to person, and it is a particular threat for nosocomial transmission, with a potentially lethal impact on health care workers [6].

The few companies newly engaged in TB drug development remain risk-averse, generally embarking on drug development only when given evidence of rigorously validated targets and lead compounds that inhibit them. The Global Alliance for TB Drug Development, a product development partnership devoted to fostering preclinical and clinical development of new TB drugs, has helped to move the few available lead compounds into development (Figure 1), but has had limited impact on early stages of TB drug discovery. Consequently, it has fallen in large part to academia to undertake early stage drug discovery. In practical terms, though, the lack of sustained funding for drug discovery and lack of access to industrial expertise and facilities, including medicinal chemistry, are major obstacles.

**Figure 1:**
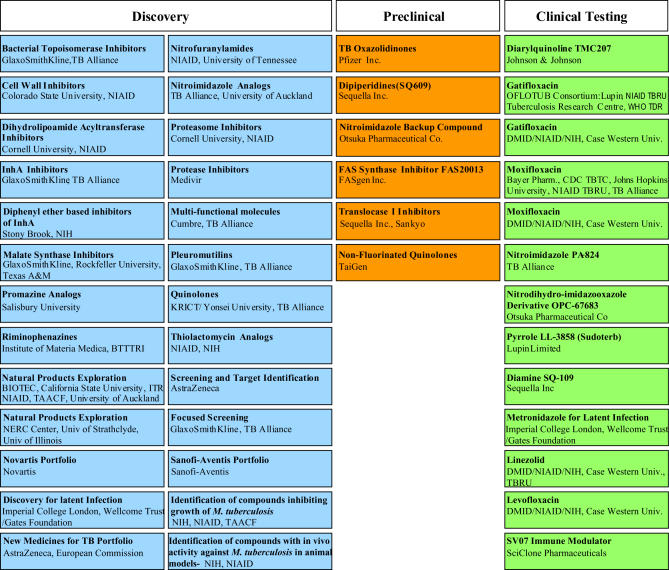
Global TB Drug Pipeline, January 2007 (Figure: Kindly provided by STOP TB Partnership Working Group on New TB Drugs) BTTTRI, Beijing Tuberculosis and Thoracic Tumor Research Institute; CDC, Centers for Disease Control and Prevention; DMID, Division of Microbiology and Infectious Diseases; ITR, Institute for Tuberculosis Research; KRICT, Korean Research Institute of Chemical Technology; NERC, Natural Environment Research Council; NIAID, National Institute of Allergy and Infectious Disease; NIH, National Institutes of Health; TAACF, Tuberculosis Antimicrobial Acquisition and Coordinating Facility; TBRU, Tuberculosis Research Unit; TBTC, Tuberculosis Trials Consortium; TDR, Special Programme for Research and Training in Tropical Diseases; WHO, World Health Organization

**Summary Points**

- For the first time in decades, there is a TB drug pipeline, but the paucity of candidates is still cause for alarm given the global emergence of drug-resistant TB
- Early-stage drug discovery represents one of the key bottlenecks in the search for new anti-TB drugs
- Scientists, clinicians, and pharmaceutical, government, and foundation representatives met in January at a symposium organized by Médecins Sans Frontières to consider urgent actions to address the roadblocks in TB drug R&D
- Participants discussed new approaches to ensure collaboration, innovation, and sustained application of academic and industry expertise to address major neglected diseases like TB
- Experts widely recognized the need to create alternative incentive mechanisms to stimulate R&D not through high pricing of medicines, but rather, by rewarding the impact of inventions on health care outcomes

While the existence of a TB drug pipeline after decades of virtually no TB drug R&D is welcome, there are still far too few compounds that represent new chemical classes with novel mechanisms of action and a low probability of encountering pre-existing drug resistance. Of the approximately 40 compounds in the current pipeline, it is unlikely that a useful therapy will emerge, given that only about one compound in 20 successfully emerges from an anti-infective drug discovery program [7]. Since new drugs for TB should only be used in combination, to prevent resistance, it would be a responsible act of global leadership to take whatever steps are necessary to attract approximately 60 new lead compounds into the pipeline as quickly as possible. Nothing less will avert the escalation of what is already a major public health catastrophe.

## Key Obstacles in Current Initiatives

Despite some important recent initiatives—most notably those supported by the United States National Institutes of Health, the European Union, and the Bill & Melinda Gates Foundation, and R&D units set up by Novartis, GlaxoSmithKline, AstraZeneca, and Sanofi-Aventis—major limitations remain. It is becoming clear that current funding paradigms and strategic approaches have not led to the increase in drug discovery activities that will be required to respond to the emergency posed by TB.

Overall funding for TB research in general, and drug discovery in particular, remains alarmingly inadequate. The fight against HIV/AIDS has been greatly fostered by the launch of specific research programs and the creation of dedicated research agencies at the national level, such as France's “Agence Nationale de Recherches sur le Sida.” In contrast, TB research is funded in competition with all other areas of biomedicine and is clearly not receiving funds commensurate with the global dimension of the disease and the probability that untreatable forms of TB will become increasingly widespread.

Another critical obstacle is the lack of access to information, pharmaceutical expertise, compounds, and research tools. There would be great value, for example, in a publicly accessible database that collected thorough information about screenings of compounds and about analyses that indicate which targets in Mycobacterium tuberculosis appear to be “druggable.” Considering the limited resources for TB drug development, it is critical to avoid repetitive efforts, particularly multiple independent journeys to a dead-end.

The attempts to build fruitful collaborations between academia and industry in drug discovery are hampered by the failure to address access considerations in the licensing agreements that underpin such collaborations. Recent proposals suggest ways to move forward. For example, Equitable Access Licensing provides an approach to commercial exploitation of scientific research results that ensures optimal access to discoveries originating from academia when derivative technologies and/or products can be used in low- and middle-income countries [8]. While there are encouraging examples of such open licensing approaches related to discoveries of importance to the developing world, the use of such licenses must become the norm. Indeed, the recent Philadelphia Consensus Statement on university policies for health-related innovations argues that universities should: “require the inclusion of licensing terms in exclusive technology transfer agreements that ensure low-cost access to health-related innovations in the developing world” [9].

Transfer of compounds from industry libraries to academia has also been complicated by legal and intellectual property issues. Some pharmaceutical companies have given universities access to small subsets of industry compound collections, but this usually requires lengthy negotiations to define legal agreements on a case-by-case basis, making the whole process too slow to allow a productive interface with university investigators.

The lack of appropriate compound libraries for anti-infectives presents yet another obstacle, for academics and industrial scientists alike. In academia, combinatorial chemical libraries are likely to be of low yield, and those that are affordable may be of low quality, or may not include access to ready re-supply, which is necessary to work up hits that appear promising in early stages.

The principal strategies that are currently used to enhance drug discovery for neglected diseases such as TB are the creation of consortia that merge academic and industrial expertise to run “virtual” drug discovery projects and the establishment of drug discovery centers in the public sector. These strategies help enhance activities in the field, and have brought some successful outcomes in finding new treatments for other neglected diseases [10,11]. Drug discovery, though, especially in the early stages, requires continuous interactive exchange among scientists from a broad range of disciplines that is not easy to reproduce on a virtual basis. Furthermore, to build drug discovery know-how in academic and public settings is a daunting task, because the recent limited increase in access to screening facilities has not been matched by the requisite access to medicinal chemistry and other pharmaceutical expertise.

## Possible Ways Forward

One possible approach for generating a significant scale-up of TB drug discovery is to improve public sector capacity for running drug discovery programs. Government funding agencies could establish a medicinal chemistry resource center that would work as a core facility offering free lead optimization and ADMET (absorption, distribution, metabolism, excretion, and toxicology) studies in animal models. Such a facility would be directed by scientists with experience in drug discovery, and could also carry out training activities in order to ensure medicinal chemistry expertise in academia.

A major challenge, though, would be attracting talented scientists to the not-for-profit medicinal chemistry sector and retaining them in competition with industry. Moreover, to invest in medicinal chemistry for the public sector without the other technologies, resources, and expertise that go into drug development might result in limited success or strategic failure.

An alternative could be for governments to create incentives for pharmaceutical and biotech companies to run in-house phenotypic screens for anti-TB drugs with all available existing compound libraries. The scientific rationale is that target-based approaches have been unsuccessful in the area of anti-infective agent discovery [7], whereas two new potential antimicrobial agents, platensimycin, and the diarylquinoline, R207910, have recently emerged from whole organism screens [12,13]. However, shortening TB chemotherapy requires the discovery of drugs able to kill dormant M. tuberculosis [14], justifying the search for new molecular targets belonging to dormancy–related metabolic pathways and the need for target-based screens. Given the urgency, both target-based screening and phenotypic screening should be pursued in parallel to increase the chances of filling the TB pipeline. Unfortunately, there is limited expertise in the private sector in working with M. tuberculosis and a shortage of the specialized facilities required, such as robotically equipped biosafety level 3 containment facilities.

The decision to invest in replicating drug discovery expertise and facilities outside industry or the identification of effective incentives for companies to step into drug discovery for neglected diseases might require further strategic thinking, as will be discussed in the next paragraph. But investing in the creation of optimized compound libraries for the discovery of anti-infectives is a realistic proposition that needs to be taken into serious consideration.

## A New Paradigm for Financing R&D

Ultimately the gaps in TB drug discovery reflect the structural limitations of the current patent system as it is applied to generate incentives for essential medical R&D. To feed and advance the drug and vaccine pipelines for diseases that offer little traditional market incentive will require a mutually reinforcing alignment of three fundamental processes: innovation, incentive, and access.

First and foremost, alternatives are needed to the current system, which encourages drug development through patent-based monopoly pricing. Such alternative incentive mechanisms could stimulate R&D not through high pricing of medicines, but rather by rewarding the impact of inventions on health care outcomes [15,16]. Talks held by the World Health Organization's Intergovernmental Working Group on a global R&D framework treaty may hold some promise in suggesting alternatives to stimulate R&D in parallel to current market incentives.

However, given the problems of limited expertise in industry in the biology of neglected diseases—and given the general lack of success in recent years for industrial efforts to develop anti-infectives in general [4]—even the addition of an effective incentive system to engage biotech and pharmaceutical companies may not, by itself, quickly fill the pipelines for TB and other neglected diseases. Thus, the development of “open-access drug discovery entities” could be a new engine for academia–industry collaborations [4,16]. Open-access drug discovery entities would help overcome the obstacles that have hampered anti-infective development in recent years by offering sites for scientists to experiment with innovative approaches and generate optimized compound libraries for screening of anti-infectives. They would provide academic scientists with easy access to essential tools and expertise, possibly resulting in the optimization and scaling up of drug discovery activities undertaken in the public sector.

In addition, an open-access strategy would offer the great advantage of sharing results at the earliest opportunity and would work toward the development of specific combination therapies. Indeed, in the open-access drug discovery entities model, government-funded contracts would make it feasible for participating companies to allow scientists from academia or biotech into designated sites where, on a fee-for-service basis, they could have access to the full suite of pharmaceutical expertise and technology required for team-based drug discovery. This would offer a crucial solution, allowing close collaboration among academic and industry scientists and eliminating drawbacks of virtual drug discovery.

Representatives of Novartis and GlaxoSmithKline have stated that their facilities in Singapore and Tres Cantos, Spain, respectively, are already open to hosting academic scientists to carry out focused collaborative projects, while AstraZeneca India is pursuing a similar policy. This interest from industry gives hope that although these companies do not yet function on an open-access basis, pilot experiments could expand on the base already in place in some pharmaceutical companies.

One of the most important functions of such open-access drug discovery entities would be to collect, generate, and house chemical libraries likely to be rich sources for anti-infective agents [16]. These libraries would feature natural products or synthetic products based on natural chemophores, collected from or inspired by a much wider range of organisms than those which typically stocked company collections before the advent of combinatorial chemistry. The anti-infective libraries could also include compounds that drug companies have already developed for infectious diseases but set aside—for example, because they lack broad-spectrum activity.

These initiatives would be funded by users and governments and managed by a portfolio management committee including pharmaceutical professionals. Its task would be to evaluate submitted projects, select them for funding, and monitor their progression.

The chief objection to open-access drug discovery entities will probably be on issues of intellectual property. Hence we recommend that the governing contracts spell out a generic policy that does not require case-by-case negotiation and that includes mandatory procedures for arbitrating disputes without litigation. Acceptance will be fostered by sharing inventorship among all who contributed. What is critical is not the ownership of intellectual property but its control, and this should be vested with the funders in order to guarantee that people who need the resulting products will have access to them.

One way to ensure that priority medical needs are met while providing economic incentives is to register resulting patents under a patent track that rewards products based on the impact they have in reducing the global burden of disease [15,17]. A critical challenge in the implementation of such a model is the establishment of a treaty-based system to create a funding pool as well as the need to set clear criteria to judge the impact of new products on the burden of disease.

Although detailed modeling is still needed to define technical and legal details and economic parameters, new paradigms for encouraging early stage drug discovery will be of paramount importance if the TB drug pipeline is to address the daunting and urgent global needs.

## Abbreviations

R&D: research and development
TB: tuberculosis

## References

[pmed-0040293-b001] Nwaka S, Ridley RG (2003). Virtual drug discovery and development for neglected diseases through public-private partnerships. Nat Rev Drug Discov.

[pmed-0040293-b002] Nwaka S, Hudson A (2006). Innovative lead discovery strategies for tropical diseases. Nat Rev Drug Discov.

[pmed-0040293-b003] Chirac P, Torreele E (2006). Global framework on essential health R&D. Lancet.

[pmed-0040293-b004] Nathan C (2004). Antibiotics at the crossroads. Nature.

[pmed-0040293-b005] Alekshun MN, Levy SB (2007). Molecular mechanisms of antibacterial multidrug resistance. Cell.

[pmed-0040293-b006] Gandhi NR, Moll A, Sturm AW, Pawinski R, Govender T (2006). Extensively drug-resistant tuberculosis as a cause of death in patients co-infected with tuberculosis and HIV in a rural area of South Africa. Lancet.

[pmed-0040293-b007] Payne DJ, Gwynn MN, Holmes DJ, Pompliano DL (2007). Drugs for bad bugs: Confronting the challenges of antibacterial discovery. Nat Rev Drug Discov.

[pmed-0040293-b008] Kapczynski A, Chaifetz S, Katz Z, Benkler Y (2005). Addressing global health inequities: An open licensing approach for university innovations. Berkeley Technol Law J.

[pmed-0040293-b009] Universities Allied for Essential Medicines (2006). Philadelphia consensus statement on university policies for health-related innovations. http://www.essentialmedicine.org/cs/?page_id=3.

[pmed-0040293-b010] Vennerstrom JL, Arbe-Barnes S, Brun R, Charman SA, Chiu FC (2004). Identification of an antimalarial synthetic trioxolane drug development candidate. Nature.

[pmed-0040293-b011] De D, Krogstad FM, Byers LD, Krogstad DJ (1998). Structure-activity relationships for antiplasmodial activity among 7-substituted 4-aminoquinolines. J Med Chem.

[pmed-0040293-b012] Andries K, Verhasselt P, Guillemont J, Gohlmann HW, Neefs JM (2005). A diarylquinoline drug active on the ATP synthase of Mycobacterium tuberculosis. Science.

[pmed-0040293-b013] Wang J, Soisson SM, Young K, Shoop W, Kodali S (2006). Platensimycin is a selective FabF inhibitor with potent antibiotic properties. Nature.

[pmed-0040293-b014] McKinney JD (2000). In vivo veritas: The search for TB drug targets goes live. Nat Med.

[pmed-0040293-b015] Pogge T (2005). Human rights and global health: A research program. Metaphilosophy.

[pmed-0040293-b016] Nathan C (2007). Aligning pharmaceutical innovation with medical need. Nat Med.

[pmed-0040293-b017] Love J (2006). Drug development incentives to improve access to essential medicines. Bull World Health Organ.

